# Nasal delivery of *Lacticaseibacillus rhamnosus* GG modulates respiratory immune responses and attenuates *Mycoplasma pneumoniae* pneumonia in a murine model

**DOI:** 10.1128/spectrum.00771-25

**Published:** 2025-11-14

**Authors:** Jiarong He, Wei Tian, Pengxiao Feng, Tingfeng Du, Huanbing Long, Guiting He, Hongjiang Wei, Xinyan Zhu, Xinyue Jiang, Cuiming Zhu

**Affiliations:** 1 Hunan Provincial Key Laboratory for Special Pathogens Prevention and Control, Institute of Pathogenic Biology, Hengyang Medical College, University of South China 34706, Hengyang, Hunan, People’s Republic of China; 2 Affiliate Nan Hua Hospital, University of South China 34706, Hengyang, Hunan, People’s Republic of China; City of Hope, Department of Pathology, Duarte, California, USA

**Keywords:** *Mycoplasma pneumoniae*, *Lactobacillus rhamnosus *GG, intranasal administration, immune regulation, respiratory immune responses

## Abstract

*Mycoplasma pneumoniae* pneumonia (MPP) management presents significant challenges due to the lack of an effective vaccine and the widespread occurrence of macrolide resistance. Recent studies investigating the use of oral or intranasal probiotics have shown promising results in reducing respiratory tract infections. In this study, BALB/c mice were initially given intranasal doses of *Lacticaseibacillus rhamnosus* GG (LGG), *Limosilactobacillus reuteri* F275, or a combination of these two strains, called MIX, to evaluate the safety of probiotic administration by examining lung tissue inflammation. Our results suggest that LGG is relatively safe, with minimal lung inflammatory responses, unlike the mild inflammation seen with *L. reuteri* F275 and MIX treatments. Next, LGG was administered to the mice via the respiratory tract on two consecutive days before or after infection with *M. pneumoniae*. The findings showed that both pre- and post-infection intranasal LGG significantly reduced lung inflammation caused by *M. pneumoniae* and decreased the pathogen load. The protective effects of nasal LGG delivery were associated with an increase in alveolar macrophages, higher levels of IgA in the bronchoalveolar lavage fluid (BALF), and increased IgG levels in the serum, all of which contribute to the elimination of the pathogen. Additionally, LGG pretreatment decreased neutrophil levels in the BALF and lungs, suppressed pro-inflammatory cytokines (tumor necrosis factor α, interleukin [IL]-6, and IL-17A), and boosted the production of the anti-inflammatory cytokine IL-10 in the BALF, reducing MPP severity. Intranasal administration of LGG appears to be a safe and potentially effective strategy for alleviating MPP in mouse models.

**IMPORTANCE:**

The increasing prevalence of antibiotic-resistant *Mycoplasma pneumoniae* pneumonia (MPP), together with the absence of an effective vaccine, highlights the critical demand for alternative therapeutic approaches. This study demonstrates that intranasal delivery of *Lacticaseibacillus rhamnosus* GG (LGG) significantly mitigates pulmonary inflammation and decreases bacterial load in a mouse model of MPP, both prophylactically and therapeutically. Notably, LGG strengthens essential immune defenses by promoting alveolar macrophage recruitment, increasing secretory IgA in the airways, and stimulating systemic IgG. It also modulates cytokine production, suppressing pro-inflammatory mediators while enhancing anti-inflammatory IL-10. These findings support intranasal LGG administration as a strategy with promising translational potential for MPP.

## INTRODUCTION

Probiotics are “live microorganisms that, when administered in adequate amounts, confer a health benefit on the host” (1). Probiotic benefits are specific to certain strains and do not apply universally to the entire genus or all its species. Research indicates that many probiotic strains provide health benefits to humans. A well-known group with recognized probiotic potential is the genus Lactobacillus, which consists of gram-positive bacteria commonly found in various parts of the human body, including the gastrointestinal, oral, vaginal, and respiratory tracts. Some specific strains within this genus are widely used as probiotics in commercial products and as fermenting agents in dairy production.

*Lacticaseibacillus rhamnosus* GG (LGG) and *Limosilactobacillus reuteri* F275 (or *Lactiplantibacillus plantarum* NCIMB 8826) are common lactobacilli that are generally considered safe (2–4). The immunomodulatory properties of LGG and *L. reuteri* F275 have garnered significant attention due to their beneficial effects on the gastrointestinal mucosa (5, 6). Multiple clinical studies have demonstrated that taking LGG orally reduces the risk or decreases the duration of respiratory infections, including those induced by the H1N1 influenza virus and *Pseudomonas aeruginosa* (7–10). *L. reuteri* F275 has been demonstrated to enhance survival rates and reduce lung viral load and pulmonary inflammation in cases of pneumonia virus infection (11, 12). Nevertheless, certain studies have indicated that Lactobacillus species, whether alive or heat-inactivated, when administered to the respiratory tract, can elicit a more effective immunomodulatory response and mitigate the symptoms of respiratory pathogenic infections or allergic diseases (13–16). According to the research conducted by Percopo CM et al., the administration of either live or heat-inactivated *L. reuteri* F275 via intranasal delivery to mice before exposure effectively mitigated pulmonary inflammation, reduced viral load, and significantly increased survival rates in mice, resulting in a significant and sustained protective effect against subsequent lethal respiratory virus infections (12). The intranasal administration of LGG was found to upregulate the activity of lung natural killer (NK) cells and to stimulate the production of pro-inflammatory cytokines, including tumor necrosis factor α (TNF-α), interleukin (IL)−1β, and monocyte chemoattractant protein-1 (MCP-1), within the pulmonary tissues of BALB/c mice. This immunological response enhanced survival rates among adult mice infected with H1N1 (13). Similarly, neonatal C57BL/6 mice exhibited significantly improved survival rates and alleviated symptoms when administered intranasal LGG, leading to elevated levels of type I IFN (16). Notably, intranasal LGG administration demonstrated greater efficacy than oral supplementation in combating influenza virus infection, and live bacteria were more effective than inactivated bacteria (14, 17).

*Mycoplasma pneumoniae* constitutes a significant etiological agent in community-acquired pneumonia, particularly among school-aged children and young adults (17, 18). In recent years, the emergence of antibiotic resistance and the lack of efficacious vaccines have led to increasingly significant treatment challenges (19, 20). Our previous studies have shown that the oral administration of the probiotics *Lacticaseibacillus casei* CNRZ1874, LGG, and *L. reuteri* F275 can mitigate *M. pneumoniae* pneumonia (MPP) (21, 22). In this study, we demonstrated that in a murine model, pre- and post-intranasal inoculation with live LGG effectively reduced pathogen loads and mitigated respiratory infections following exposure to *M. pneumoniae*. The observed effects may be attributed to the immune regulatory properties of LGG, which decrease the expression of pro-inflammatory cytokines induced by the pathogen. Furthermore, LGG appears to promote the recruitment of alveolar macrophages and enhance the production of anti-inflammatory cytokine IL-10 and mycoplasma-specific immunoglobulin IgA (IgA) in bronchoalveolar lavage fluid (BALF) and IgG in serum.

## MATERIALS AND METHODS

### Mice

BALB/c mice, 6 weeks old, were obtained from Jiangsu Jicui Yaokang Biotechnology Co., Ltd. These animals were housed in a specific pathogen-free environment at the University of South China’s animal facility. Before initiating the experiment, the mice underwent a 7-day adaptation period in their housing environment.

### Lactobacilli intranasal inoculations

The LGG (ATCC 53103) strain was generously provided by Professor Xiaohua Chen of Hengyang Normal University, China. *L. reuteri* F275 (ATCC23272, DSM20016, or CIP109823) was acquired from BeiNuo Biological Company, Shanghai, China. The LGG and *L. reuteri* F275 bacterial strains were cultivated in Man, Rogosa, and Sharpe (MRS) medium (Solarbio, China) at 37°C for 12–16 h. Subsequently, the cultures were centrifuged at 3,000 *× g* for 10 min. The supernatant was removed, and the bacterial pellet was rinsed once with sterile phosphate-buffered saline (PBS). The bacteria were then resuspended in sterile PBS to achieve a final concentration of 2.5 × 10^10^ colony-forming units (CFUs)/mL. After anesthetization with inhaled isoflurane, 10^9^ CFUs of LGG, 10^9^ CFUs of *L. reuteri* F275, or a combination of both (5 × 10^8^ CFUs of LGG and 5 × 10^8^ CFUs of *L. reuteri* F275) in 40 µL of PBS was intranasally inoculated into mice, with PBS delivery as a control. Respiration, weight loss, and locomotion were assessed daily.

### *M. pneumoniae* preparation and infection

The standard strain of *M. pneumoniae* type 1, M129 (ATCC 29342), was maintained at the Institute of Pathogen Biology, Hengyang Medical College, University of South China. This strain was cultivated in a pleuropneumonia-like organism (PPLO) medium (BD Biosciences, USA). Cultivation occurred in a 225 cm^2^ cell culture flask at 37°C until the medium’s color changed from red to orange. Subsequently, the medium was carefully removed by gentle decantation, and the bottom of the vial was gently scraped with a cell scraper for several minutes. The sample was centrifuged for 15 min at 10,000 *× g* to harvest mycoplasma cells, and the cells were washed once with sterile PBS and then resuspended in the same medium. The CFUs *of M. pneumoniae* were assessed using the following protocol: After freezing for several days, a tube of mycoplasma was retrieved from the freezer and subjected to a single rapid freeze-thaw cycle. CFUs were then determined by plating serial dilutions of the organism onto CM401 solid medium (Oxoid) and counting colonies under 100× magnification after approximately 7 days of incubation. Additional *M. pneumoniae* aliquots stored at −80°C were used for immunological testing or challenge experiments, all of which were performed within 1 month of storage. All *M. pneumoniae*-infected mice received intranasal inoculation with 10^8^ CFUs of this organism in 40 µL of PBS under isoflurane anesthesia. Mice were euthanized at 3 and 7 days post-infection (dpi) via carbon dioxide inhalation. The 3 dpi time point was selected to assess LGG’s protective efficacy against an acute *M. pneumoniae* challenge and its modulation of innate immunity, specifically neutrophil and macrophage activation (23, 24). The 7 dpi time point was chosen to evaluate the adaptive immune response, characterized by significant increases in pathogen-specific IgM antibodies (22, 24) and markedly elevated lung T-cell counts, as previously demonstrated. Cardiac blood, BALF, and lung samples were collected for further analysis.

### The number of *M. pneumoniae* colonies

The collected BALF was centrifuged at 900 × *g* for 10 min. Subsequently, 50 µL of the resulting supernatant was uniformly distributed on a solid PPLO medium. The prepared medium was then placed in a temperature-controlled incubator maintained at 37℃, with an atmosphere of 95% N2 and 5% CO2, for 7–10 days. The solid plate was then placed under a standard inverted microscope for observation, and the number of mycoplasma colonies was quantified under low magnification.

### Histopathology scoring

The right lower lobe of the lung was fixed in a 4% paraformaldehyde solution (Biosharp, China) for 48 h. Subsequently, paraffin embedding, sectioning, and hematoxylin and eosin staining were performed, and pathological inflammatory damage to the lung tissue was observed utilizing a double-blind method. Histopathology scoring (HPS) was assessed by analyzing the quantity and infiltration area of inflammatory cells in the alveoli and bronchi, the extent of proliferation of blood vessels and bronchial tube walls, and the delineation of borders (25).

### Lactate dehydrogenase production

Following the manufacturer’s protocol, lactate dehydrogenase (LDH) production in the BALF supernatant, an indicator of lung tissue cytotoxicity, was quantified using an LDH assay kit (Solarbio, China).

### Cell sorting and counting

200 µL of cardiac blood was transferred to an EDTA-K2 anticoagulant tube and gently inverted to ensure thorough mixing. Cell analysis was performed using an XN-1000V Animal Hematology Analyzer (Xisenmecan).

Cells in BALF were isolated by centrifugation at 900 × *g* for 10 min, after which they were resuspended in PBS for subsequent analysis. Lung tissue specimens were excised and homogenized in 1 mL of sterile PBS. The homogenate was filtered through a 40-micron cell strainer and then centrifuged at 1,200 *× g* for 5 min. The resultant pellet was resuspended in a 35% PERCOLL solution and subjected to density gradient centrifugation at 700 × *g* for 15 min. The upper liquid and impurities were carefully removed. Subsequently, ACK lysis buffer was applied to the sediment to lyse erythrocytes at room temperature for 5 min. Finally, pulmonary cells were harvested by centrifugation at 1200 × *g* for 5 min (26). The cells from BALF and pulmonary tissue were then stained with anti-mouse CD45-FITC, anti-mouse Ly-6G-PE, anti-mouse CD11c-PerCP-Cy5.5, and anti-mouse Siglec-F-A647(APC) antibodies. Following immunostaining, cells were analyzed using flow cytometry. Specifically, CD45+ cells were identified as leukocytes, CD45+Ly6G+ cells as neutrophils, and CD45+CD11c+Siglec-F+ cells as alveolar macrophages. Absolute count of pulmonary neutrophils or macrophages = total cells in the sample × percentage of target cells (%).

### Cytokines in BALF

Under the manufacturer’s protocol, an indirect enzyme-linked immunosorbent assay (ELISA) was conducted to quantify the cytokines TNF-α, IL-6, IL-10, IL-17A, and transforming growth factor-beta (TGF-β) in the supernatant obtained from BALF. The absorbance was measured at A450, and the concentrations of the cytokines were calculated based on the established standard curve.

### Reverse transcription-quantitative PCR

The total RNA from pulmonary cells was isolated using TRIzol reagent (TIANGEN, China) and subsequently converted to cDNA using FastKing One-Step Genomic cDNA First-Strand Synthesis Premix Reagent (KR118) (TIANGEN, China). Subsequently, the *Muc5a* gene was detected through quantitative reverse transcription PCR (qRT-PCR) utilizing a SYBR Green Premix Ex Taq kit (Bio-Rad, 1725121). The analysis was conducted using the 2^−ΔΔCt^ method, with β-actin as the internal control. The sequences of the primers are presented as follows: *Muc5a* forward, 5′-CAGGACTCTCTGAAATCGTACCA-3′; *Muc5a* reverse, 5′-AAGGCTCGTACCACAGGGA-3′; β-actin forward, 5′-TGCTGTCCCTGTATGCCTCT-3′; β-actin reverse, 5′-AGGTCTTTACGGATGTCAACG-3.'

### Intracellular cytokines and Foxp3

Pulmonary cells were stimulated with 50 ng/mL PMA, 1 µg/mL ionomycin, and 1 µmol/mL brefeldin A for 4 h. After which, the cells were blocked with rat anti-mouse Fc receptor (CD16/CD32) antibody (BD Biosciences), and then incubated with anti-mouse CD3e-PE-Cy7 (BD PharMingen, USA), anti-mouse CD4-PE (BioLegend, USA), and anti-mouse CD8a-FITC (BioLegend) for 30 min at 4°C for surface molecules immunostaining. Subsequently, dead cells were distinguished by staining with the anti-mouse dye eflour@560. Following this, the cells were fixed and permeabilized using a buffer set (BD Biosciences) and subsequently incubated with anti-mouse IFN-γ-APC and anti-mouse IL-4-PerCP-Cy5.5 (BioLegend, USA) for 30 min. For Treg analysis, pulmonary cells were stained with rat anti-mouse Fc receptor (CD16/CD32) antibody and anti-mouse CD4-PE. After fixation and permeabilization, cells were incubated with anti-mouse Foxp3-Alexa Fluor (APC) (BD PharMingen, USA) for 30 min. After staining, the cells were collected by centrifugation at 12,000 × *g* for 5 min and resuspended in FACS buffer. Finally, the samples were examined using an LSRII flow cytometer (BD Biosciences), and the resulting data were analyzed using FlowJo software. The cells underwent two washes at each procedural step using a fluorescence-activated cell sorting (FACS) buffer; however, sterile PBS was used for washing before staining the dead cells with the anti-mouse dye eFlour@560.

### Antibodies in serum and BALF

To prepare the antigen, *M. pneumoniae* was collected and centrifuged at 10,000 × *g* for 15 min. Next, the cells were washed with sterile PBS before being harvested by centrifugation. The resulting pellet was suspended in 10 mL of PBS and disrupted using ultrasonic waves. The sonication process consisted of 3-second bursts followed by 10-second intervals, continuing for 1 h. The protein content of the samples was determined by a BCA kit (Epizyme, China).

ELISA was used to detect the production of *M. pneumoniae*-specific serum IgG and BALF IgA. In summary, *M. pneumoniae* antigens were suspended in a carbonate coating buffer at a concentration of 50 mmol/L, with a pH of 9.6, to achieve a final concentration of 15 µg/mL. The antigen solution was subsequently applied to 96-well microtiter plates and incubated overnight at 4°C. Following incubation, the plates were thoroughly washed with buffer and blocked with 200 µL of 5% skim milk powder for 2 h at 37°C. After an additional washing step, 100 µL of diluted serum or BALF supernatant was added to the designated test wells, and the plates were incubated for a further 2 h at 37°C. The plates were rewashed, after which horseradish peroxidase-conjugated goat anti-mouse IgG or IgA antibodies (Proteintech, USA) were added at dilutions of 100 µL per well. The plates were incubated for 1 h at 37°C. Finally, 100 µL of TMB solution was added to each test well, and the plates were incubated in the dark at 37°C for 15 min. The chromogenic reaction was halted with a stop solution, and the optical density was measured using an ELISA instrument (PerkinElmer, USA) to quantify IgG and IgA levels.

### Statistical analyses

Statistical analyses were performed using GraphPad Prism 8 software (GraphPad Software, Inc., La Jolla, CA, USA). Results are presented as mean ± standard deviation (SD). Differences were assessed using a two-way ANOVA with the Sidak multiple comparison test or a Student’s t-test. Statistical significance was defined as *P* < 0.05.

## RESULTS

### Impact of nasal delivery of Lactobacillus on mouse pulmonary tissue

**Fig 1:**
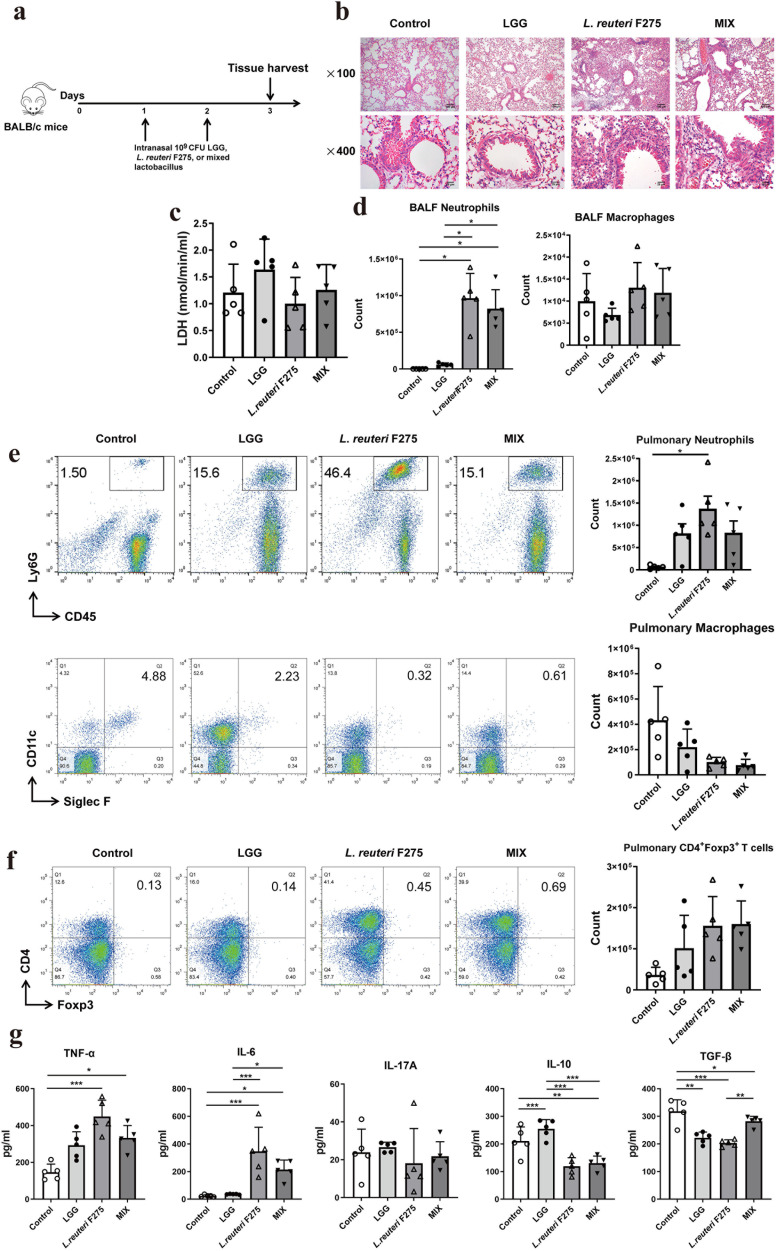
Effects of nasal administration of Lactobacillus in mice lungs. On the first day following Lactobacillus supplementation, mice were sacrificed, and the lungs and BALF were collected for further analysis. (**a**) Experimental procedure for intranasal Lactobacillus supplementation in mice. (**b**) Lung histopathology (representative images at ×100 and ×400 magnification for each experimental group). (**c**) LDH content in BALF. (**d and e**) Neutrophils and alveolar macrophages were detected and quantified in both lung tissue and BALF using flow cytometry. (**f**) CD4+Foxp3+ cells in the mouse lung tissue were analyzed by flow cytometry. (**g**) Secretion of pro-inflammatory cytokines in the BALF was quantified using ELISA. *N* = 20, *n* = 5. Each value indicates mean ± SD. * *P* < 0.05, ** *P* < 0.01, *** *P* < 0.001.

To evaluate the safety of intranasal Lactobacillus administration, alterations in the lung tissues of mice were examined. The experimental procedure was illustrated in Fig. 1a. Although the alveolar structure remained relatively intact, the number of inflammatory cells and area of infiltration of inflammatory cells into the alveoli of mice intranasally administered live *L. reuteri* F275 and MIX increased (Fig. 1b). Although no significant differences were observed in LDH production in BALF or the population of regulatory T cells (Tregs) in the lungs of mice inoculated intranasally with PBS, LGG, *L. reuteri* F275, and MIX (Fig. 1c and g), the MIX formulation and PBS control, nasal administration of *L. reuteri* F275, and the MIX formulation significantly increased neutrophil counts within pulmonary tissue and elevated levels of TNF-α and IL-6 in BALF in the murine model (Fig. 1b through g). At the same time, a reduction in IL-10 and TGF-β production was observed (Fig. 1g). Conversely, LGG administration enhanced IL-10 secretion without affecting neutrophil influx or the levels of TNF-α, IL-6, and TGF-β (Fig. 1g).

### Pre-intranasal administration of LGG alleviated MPP

**Fig 2:**
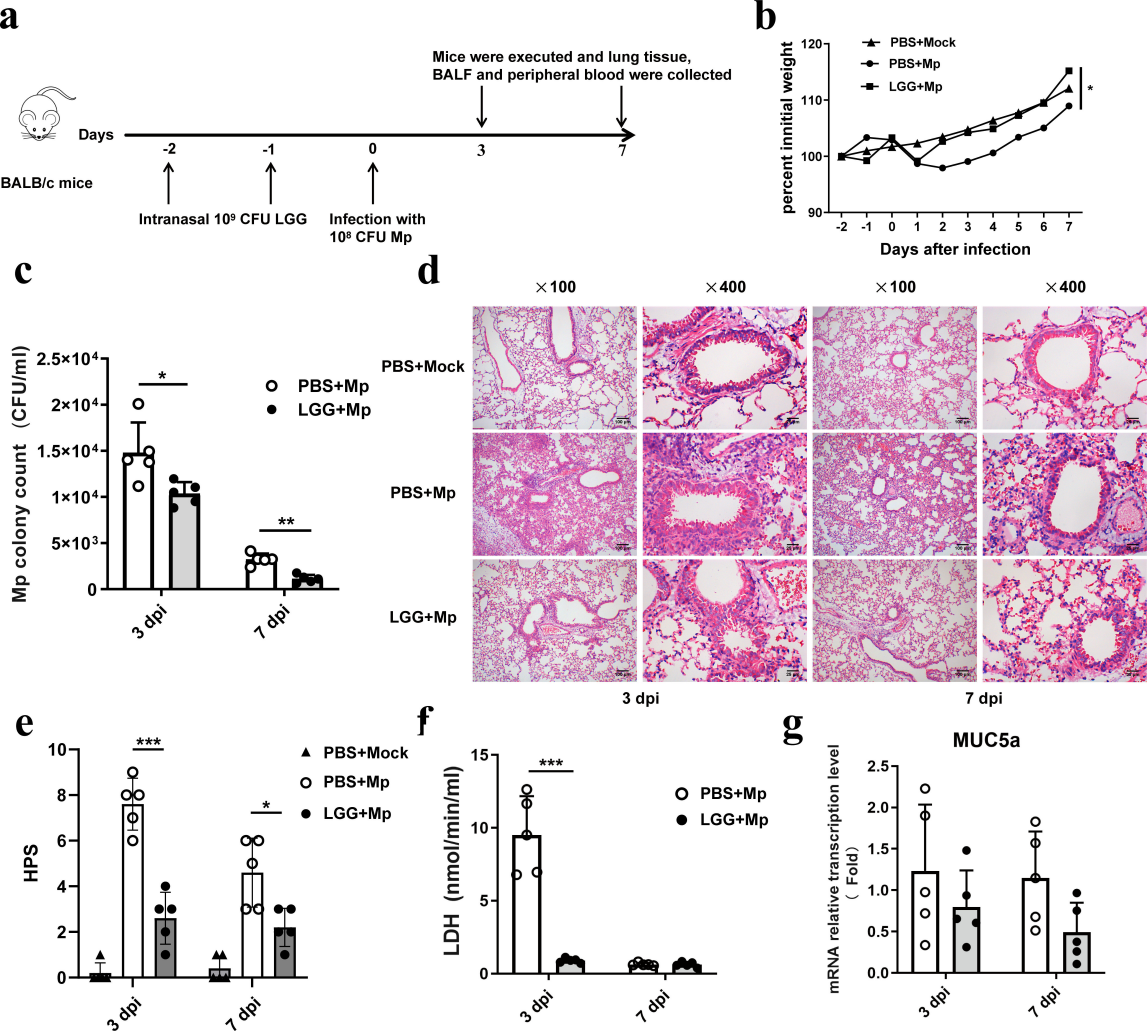
LGG pre-treatment reduces lung tissue damage caused by *M. pneumoniae* infection. Mice were nasally pre-administered with LGG and then infected with *M. pneumoniae* via the respiratory tract. The animals were euthanized at 3 and 7 dpi. (**a**) Experimental procedure for nasal pretreatment with Lactobacillus before *M. pneumoniae* infection, (**b**) Body weight, (**c**) *M. pneumoniae* colony counts in mouse BALF, (**d**) lung histopathology (representative images at ×100 and ×400 magnification for each experimental group), (**e**) HPS, (**f**) LDH content in BALF, and (**g**) mRNA relative transcription of MUC5a in mouse lungs, *n* = 5. Each value shows the mean ± SD. * *P* < 0.05, ** *P* < 0.01, *** *P* < 0.001.

The mycoplasma cell load and inflammatory pathology in the lungs of mice were determined to evaluate the effect of the pre-intranasal administration of LGG on *M. pneumoniae* infection. The experimental design is illustrated in Fig. 2a. Intranasal administration of LGG before the infection with *M. pneumoniae* did not yield a statistically significant difference in the weight of the mice when compared to the control group (Fig. 2b). Three days after infection, pre-treatment with LGG substantially lowered the pathogen burden (Fig. 2c). Additionally, a reduced presence of inflammatory cells, smaller areas of infiltration, and more intact alveolar structures were observed in the lungs of LGG-administered mice, along with a significant decrease in HPS and LDH compared to the PBS pre-treated control group (Fig. 2d through f), providing further evidence for the mitigation of lung tissue damage. MUC5a is upregulated in response to inflammatory conditions, and it has been reported that excessive production of MUC5a is a major contributor to airway obstruction. It was reported that probiotic and prebiotic therapy could significantly reduce the transcription of the *MUC5a* gene in asthmatic mice (27, 28); however, our study showed that the administration of LGG did not alter the transcription levels of *MuC5a* in the lungs (Fig. 2g). Seven days post-infection, the LGG-pretreated group demonstrated a reduction in *M. pneumoniae* burden compared to the control group, whereas no significant differences were observed between the two groups regarding pulmonary histopathology, inflammatory cell counts, HPS, and MUC5a expression (Fig. 2c through g).

### Pre-treatment with LGG altered innate cellular profiles and cytokine responses

**Fig 3:**
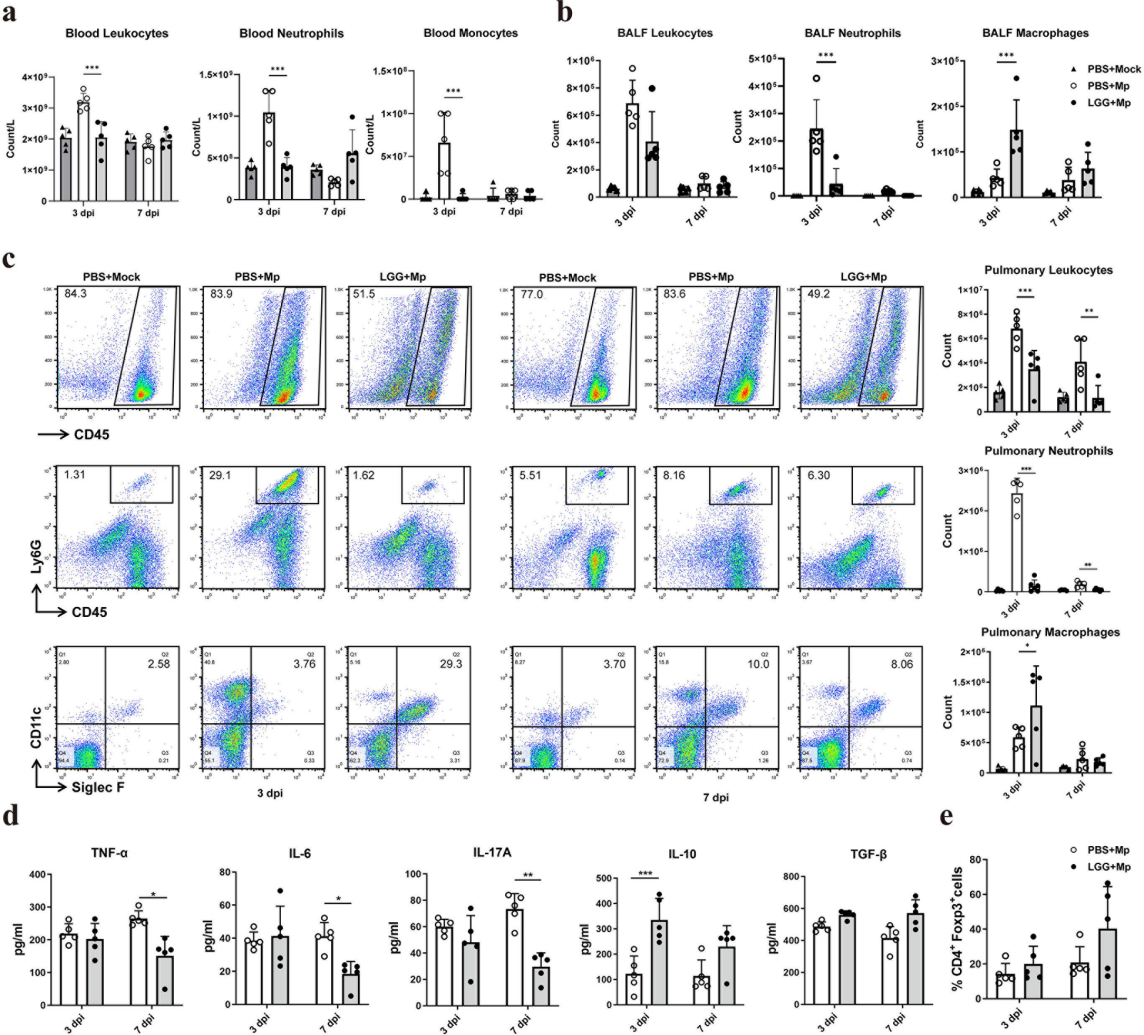
Intranasal pre-treatment with LGG altered innate cellular profiles and cytokine responses following *M. pneumoniae* infection. Mice were pre-administered LGG nasally and then subjected to *M. pneumoniae* infection via the respiratory tract. The animals were euthanized at 3 and 7 dpi. (**a–c**) Leukocytes, neutrophils, and monocytes (or alveolar macrophages) were analyzed and quantified in peripheral blood, BALF, and pulmonary cells by flow cytometry. (**d**) Cytokine concentrations in BALF were determined using ELISA. (**e**) The percentage of pulmonary Tregs was assessed by flow cytometry. *N* = 30, *n* = 5. Each value represents the mean ± SD. * *P* < 0.05, ** *P* < 0.01, *** *P* < 0.001.

To investigate the effects of intranasal LGG on the modulation of innate immunity in the context of mitigating MPP, this study analyzed the classification and enumeration of cells in the peripheral blood, BALF, and lung tissue, as well as the expression of cytokines in BALF of mice infected with *M. pneumoniae*. Three days after infection with *M. pneumoniae*, the intranasal administration of LGG led to a significant decrease in the total leukocyte count, along with reductions in the numbers of neutrophils and monocytes in the peripheral blood (Fig. 3a). In addition, there was a decrease in leukocytes and neutrophils and an increase in alveolar macrophages in the lung tissue of mice (Fig. 3a). In the BALF, the changes in leukocytes, neutrophils, and alveolar macrophages mirrored those in the lungs (Fig. 3b). Seven days after *M. pneumoniae* infection, mice that received LGG pre-treatment showed a significant decrease in lung leukocytes and neutrophils, as well as neutrophils in BALF, compared to those pretreated with PBS (Fig. 3c). On day 3 post-infection, nasal administration of LGG before *M. pneumoniae* infection increased the anti-inflammatory cytokine IL-10. At the same time, there were no changes in the levels of TNF-α, IL-6, and IL-17 (Fig. 3d). However, on day 7 post-infection, levels of the pro-inflammatory cytokines TNF-α, IL-6, and IL-17A significantly decreased (Fig. 3d). No significant differences were observed in TGF-β levels or the percentage of Tregs between the two experimental groups on days 3 and 7 post-infection (Fig. 3e).

### Pre-treatment with LGG regulated the adaptive immunity triggered by *M. pneumoniae*

**Fig 4:**
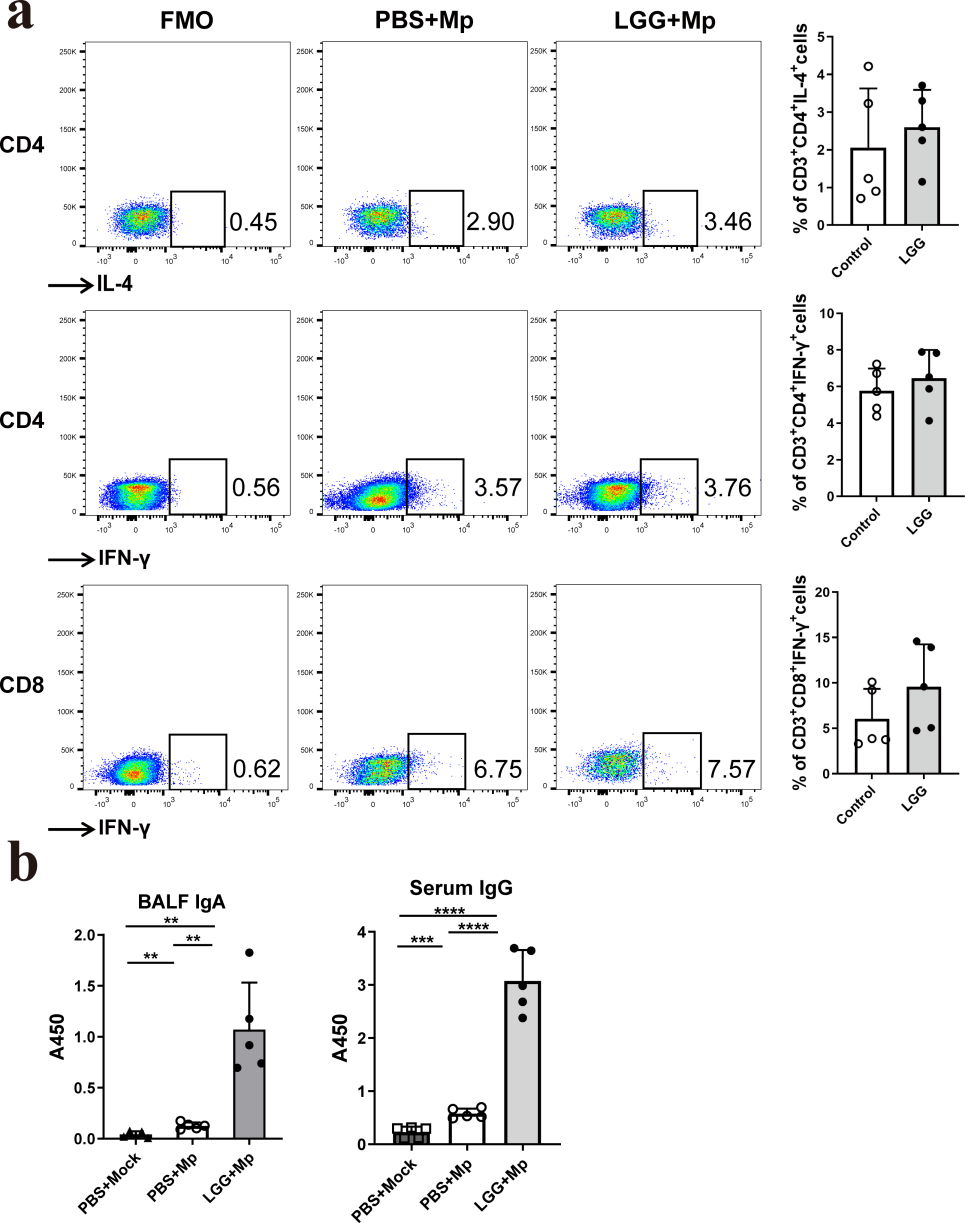
Intranasal pretreatment with LGG regulated adaptive immunity triggered by *M. pneumoniae*. Mice were pre-administered LGG nasally and then subjected to *M. pneumoniae* infection via the respiratory tract. The animals were euthanized at 7 dpi. (**a**) The percentage of T cells was analyzed by flow cytometry, and (**b**) the levels of *M. pneumoniae*-specific IgA in BALF and IgG in serum were analyzed using ELISA. *N* = 15, *n* = 5. Each value represents the mean ± SD. **P < 0.01, *** *P* < 0.001, ****P < 0.0001.

This study examined the impact of nasal pre-administration of LGG on the adaptive immune response in mice infected with *M. pneumoniae*. To gain insights into these effects, the activation of T cells and the production of specific antibodies were analyzed. The results of our study indicated that the pre-administration of LGG did not significantly alter the profiles of specific immune cells in the pulmonary tissue of infected mice. This includes immune cell populations such as CD3+CD4+IL-4+, CD3+CD4+IFN-γ+, and CD8+IFN-γ+ cells (Fig. 4a). Nevertheless, LGG resulted in a significant increase in pathogen-specific IgA levels in the BALF and enhanced IgG levels in the serum (Fig. 4b). These findings demonstrate that LGG effectively modulates the humoral immune response.

### Post-treatment with LGG enhanced the resistance to *M. pneumoniae*

**Fig 5:**
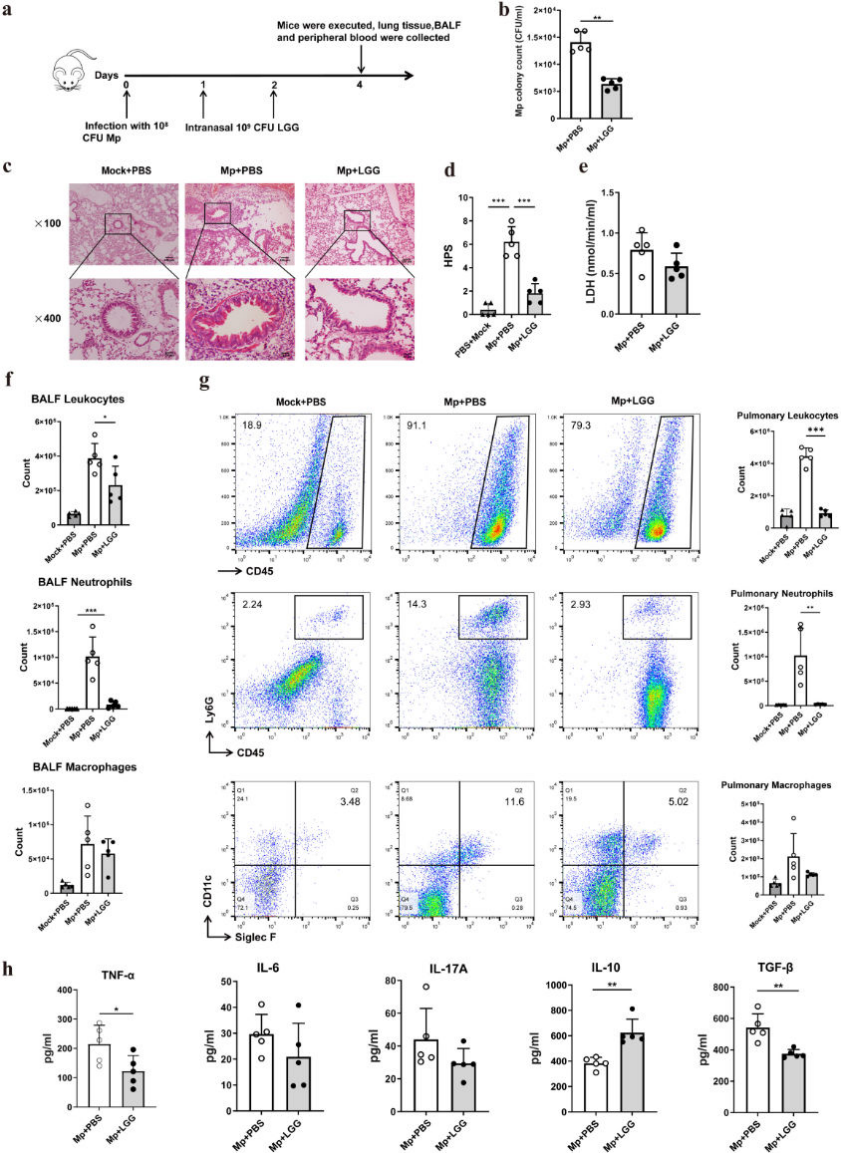
Post-treatment with LGG enhanced resistance to *M. pneumoniae*. 24 h following intranasal infection with *M. pneumoniae*, the mice were nasally treated with LGG for two consecutive days and subsequently sacrificed. (**a**) Experimental procedure for the LGG treatment, (**b**) the *M. pneumoniae* colony counts in mouse BALF, (**c**) lung histopathology (representative images at ×100 and ×400 magnification for each experimental group), (**d**) HPS, (**e**) LDH content in BALF, (**f and g**) Flow cytometry detection of leukocytes, neutrophils, and alveolar macrophages in BALF and lung tissue, (**h**) Cytokines secretion in the BALF quantified using ELISA. *N* = 15, *n* = 5. Each value represents the mean ± SD. *P < 0.05, **P < 0.01, ***P < 0.001.

To evaluate the potential of LGG as an adjunct therapy for MPP, intranasal administration was performed following *M. pneumoniae* infection. The experimental protocol is illustrated in Fig. 5a. Intranasal LGG treatment after *M. pneumoniae* infection significantly decreased pathogen colonies compared to the control group (Fig. 5b). Participants who received LGG exhibited reduced inflammatory infiltration in the peribronchial and perivascular regions, along with a decrease in HPS (Fig. 5c and d), while there was no effect on LDH levels (Fig. 5e). Additionally, LGG administration markedly lowered total leukocyte and neutrophil counts in lung tissue and BALF; however, alveolar macrophage populations remained unchanged (Fig. 5f and g). Cytokine analysis revealed that LGG therapy decreased TNF-α and TGF-β secretion and increased IL-10 production in BALF (Fig. 5h).

## DISCUSSION

Although LGG and *L. reuteri* F275 are generally considered safe intestinal probiotics in the gastrointestinal tract, their non-native status in the respiratory tract warrants examination of histopathological changes following intranasal administration. Previous research has evidenced that 24 hours following intranasal administration, *L. reuteri* F275 is no longer detectable in lung tissue and is associated with temporary, mild, heterogeneous inflammation (11). Our results revealed that intranasal administration of 10^9^ CFUs of *L. reuteri* F275 or MIX resulted in an elevated presence of neutrophils in lung tissue and the upregulation of pro-inflammatory cytokines TNF-α and IL-6 and led to minor pulmonary damage, which aligns with findings from previous research (11). In contrast to the effects observed in the lungs of mice treated with *L. reuteri* F275, our study indicates that intranasal administration of LGG is a relatively safe inoculation method. The treatment did not significantly alter the number of immune cells, cytokine release, or LDH production in the lung tissues of BALB/c mice. Furthermore, it did not cause any inflammatory pathological damage to the lung tissue. These findings are consistent with prior studies in this field (11, 12, 14, 16).

*M. pneumoniae* infection is principally responsible for developing bronchitis, tracheitis, and pneumonia (18). Although the pathogenesis of *M. pneumoniae* infection remains to be elucidated, neutrophil recruitment and the induction of pro-inflammatory and other cytokines under both acute and chronic conditions are essential components of this infection (18, 29). Accordingly, the primary objective in preventing MMP is to diminish the burden on the organism and the ensuing inflammatory response. The results of our study demonstrated that intranasal administration, either before or after infection, effectively reduced the number of mycoplasma cells and mitigated MPP in a murine model. These effects were comparable to those observed when LGG was administered orally, whether in its viable form or after heat inactivation (22).

The clearance of pathogens following *M. pneumoniae* infection primarily depends on alveolar macrophage recruitment and activation, as murine models with elevated alveolar macrophage populations are essential for resistance to this pathogen (29–31). Conversely, rather than facilitating *M. pneumoniae* clearance, excessive neutrophil presence can result in severe tissue damage (29, 32, 33), and the suppression of neutrophil itaconate production can mitigate MPP (34). Furthermore, in the development and progression of MPP, particularly in refractory cases, the equilibrium between Th17 and Treg cells plays a crucial role (35). IL-17 plays a pivotal role in *M. pneumoniae* infections and is a significant predictor of refractory MPP in pediatric patients (36). The abnormal secretion of IL-17 in pulmonary tissue can trigger the activation of epithelial cells to produce chemokines, leading to ongoing neutrophil influx, tissue damage, and potentially asthma (37–39). In contrast to Th17 cells, Tregs are generally recognized as potent suppressors of mycoplasma respiratory infections, and Tregs and IL-10 are involved in suppressing IL-17A production (40). During the acute phase of MPP, Treg functionality is notably compromised. Curiously, as the condition progresses to the resolution stage, the impairment of Treg function coincides with an elevation in Th17 cell numbers (41). The Th17/Treg ratio strongly correlates with the severity of MPP and plays a significant role in the pathogenesis of refractory MPP (40, 41).

LGG modulates innate immunity through TLR interactions, the induction of antimicrobial peptides, and the strengthening of the epithelial barrier. It influences the maturation of dendritic cells, the polarization of macrophages, and the function of neutrophils, while promoting anti-inflammatory effects. Competitive exclusion and immunomodulatory components further reduce bacterial load (42). The investigation demonstrated that mice pretreated with LGG before *M. pneumoniae* infection exhibited an increased population of alveolar macrophages, suggesting enhanced clearance of the pathogen. Furthermore, LGG pretreatment resulted in reduced leukocyte and neutrophil counts in both peripheral blood and lung tissue, along with lower levels of the pro-inflammatory cytokines IL-17A, TNF-α, and IL-6. Concurrently, IL-10 levels were elevated in the lungs, indicating reduced pulmonary inflammation. Although LGG pretreatment could reduce *M. pneumoniae* load in the lungs of mice, the decrease was not substantial, as the bacterial count in the lungs did not change by even a logarithmic unit. One reason might be that, although LGG administration can activate immune cells such as macrophages, this activation was not strong enough to produce a robust immune response that clears the pathogen. Additionally, on day 3 post-infection, the levels of pro-inflammatory cytokines in BALF were similar between mice pretreated with LGG and those with PBS, indicating an inadequate immune response to suppress and eliminate the pathogen. Furthermore, in the experiment, the interval between LGG administration and infection was short (only 2 days). LGG may not have had sufficient time to multiply and exert its immunomodulatory effects before the pathogen attack, thus failing to reduce the pathogen load significantly.

Although LGG treatment post-infection did not significantly affect alveolar macrophage populations, it decreased the number of mycoplasma colonies. This outcome may be attributed to reduced neutrophil infiltration, decreased TNF-α production, and increased IL-10 expression in the lungs. Earlier studies have shown that the oral administration of LGG results in an increase in lung Tregs in mice infected with *P. aeruginosa* (8); however, the present study did not observe an increase in Tregs or the expression of TGF-β following intranasal administration of LGG in mice infected with M. pneumoniae. Interestingly, administering LGG to mice, whether alone or following an infection with *M. pneumoniae*, resulted in a significant reduction in TGF-β secretion in the BALF. To build on these findings, further research is required to investigate the expression of the CD25 molecule in Tregs. Moreover, the processes responsible for producing Tregs and TGF-β remain unclear and require further investigation.

Undoubtedly, the course and development of *M. pneumoniae* infection are significantly modulated by adaptive immune responses. In addition to their critical role in controlling or preventing MPP, antibodies and effector T cells also inhibit the dissemination of mycoplasma infections from mucosal regions to other bodily tissues, which can potentially result in arthritis and other diseases. Earlier investigations have suggested that IgA may play a crucial role in suppressing *M. pneumoniae*’s ability to adhere during infection. IgG is vital in the body’s immune defense mechanism, particularly in the later phases of *M. pneumoniae* infections (25, 43). Our research revealed that intranasal pre-administration of LGG did not alter the number of pulmonary CD4+IL-4+ T cells, CD4+IFN-γ+ T cells, or C8+IFN-γ+ T cells in *M. pneumoniae*-infected mice. Nonetheless, our results showed that intranasal administration of LGG resulted in a significant elevation of IgA concentrations in the BALF and IgG levels in the serum. This effect may be linked to the increased number of alveolar macrophages, which act as antigen-presenting cells, enhancing the ingestion, processing, and presentation of *M. pneumoniae* antigens and subsequently activating T cells to stimulate humoral immunity. The roles of *M. pneumoniae* adaptive immunity present a dichotomy. Despite immune responses being advantageous in preventing infection, they often fail to eliminate mycoplasma and disease, resulting in the development of chronic inflammation (25, 43, 44). Furthermore, animal studies have demonstrated that the severity of the disease is exacerbated by the antibody response (45). Consequently, additional research is necessary to elucidate the impact of the heightened antibody levels induced by intranasal pre-administration of LGG.

To summarize, our findings suggest that intranasal administration of LGG is a promising alternative approach for preventing and managing MPP. Intranasal administration of LGG for two consecutive days before *M. pneumoniae* infection or 24 hours post-pathogen exposure significantly reduced the mycoplasma cell burden and ameliorated lung tissue damage in a murine model. The modulation of intranasal LGG may be responsible for this outcome, as it influences the innate and adaptive immune responses within the respiratory system when triggered by *M. pneumoniae*. These responses include increased alveolar macrophage counts, IL-10 production, and specific mycoplasma BALF IgA and serum IgG levels, concurrent with reduced neutrophil infiltration and decreased production of pro-inflammatory cytokines, such as TNF-α, IL-6, and IL-17A. However, additional research is necessary to fully understand the effects of intranasal LGG administration on *M. pneumoniae* infection, including studies with larger sample sizes, diverse animal models, and investigations into the long-term protective effects.

### Conclusion

Our results demonstrated that both intranasal pre- and post-administration of LGG significantly ameliorated the inflammatory pathology of lung tissue induced by *M*. infection and reduced the pathogen burden. The protective effects were attributed to the immunomodulatory response of nasal LGG, which involved the recruitment of leukocytes, the release of cytokines, and the production of specific antibodies. Intranasal administration with LGG is a potentially productive and safe intervention for mitigating MPP.

## Data Availability

Data will be made available on request.

## References

[1] Borchers AT, Selmi C, Meyers FJ, Keen CL, Gershwin ME. 2009. Probiotics and immunity. J Gastroenterol 44:26–46. doi:10.1007/s00535-008-2296-0 19159071

[2] O’Callaghan J, O’Toole PW. 2013. Lactobacillus: host-microbe relationships. Curr Top Microbiol Immunol 358:119–154. doi:10.1007/82_2011_187 22102141

[3] Heeney DD, Gareau MG, Marco ML. 2018. Intestinal Lactobacillus in health and disease, a driver or just along for the ride? Curr Opin Biotechnol 49:140–147. doi:10.1016/j.copbio.2017.08.004 28866243 PMC5808898

[4] Du T, Lei A, Zhang N, Zhu C. 2022. The beneficial role of probiotic Lactobacillus in respiratory diseases. Front Immunol 13:908010. doi:10.3389/fimmu.2022.908010 35711436 PMC9194447

[5] Vaarala O. 2003. Immunological effects of probiotics with special reference to lactobacilli. Clin Exp Allergy 33:1634–1640. doi:10.1111/j.1365-2222.2003.01835.x 14656348

[6] Kalyuzhin OV, Afanasyev SS, Bykov AS. 2016. Probiotics as stimulators of immune response against pathogens in the respiratory tract. Ter Arkh 88:118–124. doi:10.17116/terarkh2016885118-124 27458629

[7] Kawase M, He F, Kubota A, Harata G, Hiramatsu M. 2010. Oral administration of lactobacilli from human intestinal tract protects mice against influenza virus infection. Lett Appl Microbiol 51:6–10. doi:10.1111/j.1472-765X.2010.02849.x 20438618

[8] Khailova L, Baird CH, Rush AA, McNamee EN, Wischmeyer PE. 2013. Lactobacillus rhamnosus GG improves outcome in experimental Pseudomonas aeruginosa pneumonia: potential role of regulatory T cells. Shock 40:496–503. doi:10.1097/SHK.0000000000000066 24240593 PMC5592098

[9] Kumpu M, Kekkonen RA, Kautiainen H, Järvenpää S, Kristo A, Huovinen P, Pitkäranta A, Korpela R, Hatakka K. 2012. Milk containing probiotic Lactobacillus rhamnosus GG and respiratory illness in children: a randomized, double-blind, placebo-controlled trial. Eur J Clin Nutr 66:1020–1023. doi:10.1038/ejcn.2012.62 22692023

[10] Hojsak I, Snovak N, Abdović S, Szajewska H, Misak Z, Kolacek S. 2010. Lactobacillus GG in the prevention of gastrointestinal and respiratory tract infections in children who attend day care centers: a randomized, double-blind, placebo-controlled trial. Clin Nutr 29:312–316. doi:10.1016/j.clnu.2009.09.008 19896252

[11] Garcia-Crespo KE, Chan CC, Gabryszewski SJ, Percopo CM, Rigaux P, Dyer KD, Domachowske JB, Rosenberg HF. 2013. Lactobacillus priming of the respiratory tract: heterologous immunity and protection against lethal pneumovirus infection. Antiviral Res 97:270–279. doi:10.1016/j.antiviral.2012.12.022 23274789 PMC3608699

[12] Percopo CM, Rice TA, Brenner TA, Dyer KD, Luo JL, Kanakabandi K, Sturdevant DE, Porcella SF, Domachowske JB, Keicher JD, Rosenberg HF. 2015. Immunobiotic Lactobacillus administered post-exposure averts the lethal sequelae of respiratory virus infection. Antiviral Res 121:109–119. doi:10.1016/j.antiviral.2015.07.001 26145728 PMC4536168

[13] Harata G, He F, Hiruta N, Kawase M, Kubota A, Hiramatsu M, Yausi H. 2010. Intranasal administration of Lactobacillus rhamnosus GG protects mice from H1N1 influenza virus infection by regulating respiratory immune responses. Lett Appl Microbiol 50:597–602. doi:10.1111/j.1472-765X.2010.02844.x 20438620

[14] Youn HN, Lee DH, Lee YN, Park JK, Yuk SS, Yang SY, Lee HJ, Woo SH, Kim HM, Lee JB, Park SY, Choi IS, Song CS. 2012. Intranasal administration of live Lactobacillus species facilitates protection against influenza virus infection in mice. Antiviral Res 93:138–143. doi:10.1016/j.antiviral.2011.11.004 22120759

[15] Pellaton C, Nutten S, Thierry AC, Boudousquié C, Barbier N, Blanchard C, Corthésy B, Mercenier A, Spertini F. 2012. Intragastric and intranasal administration of Lactobacillus paracasei NCC2461 modulates allergic airway inflammation in mice. Int J Inflam 2012:686739. doi:10.1155/2012/686739 22762009 PMC3382844

[16] Kumova OK, Fike AJ, Thayer JL, Nguyen LT, Mell JC, Pascasio J, Stairiker C, Leon LG, Katsikis PD, Carey AJ. 2019. Lung transcriptional unresponsiveness and loss of early influenza virus control in infected neonates is prevented by intranasal Lactobacillus rhamnosus GG. PLoS Pathog 15:e1008072. doi:10.1371/journal.ppat.1008072 31603951 PMC6808501

[17] Edens C, Clopper BR, DeVies J, Benitez A, McKeever ER, Johns D, Wolff B, Selvarangan R, Schuster JE, Weinberg GA, et al.. 2024. Notes from the field: reemergence of Mycoplasma pneumoniae infections in children and adolescents after the COVID-19 pandemic, United States, 2018-2024. MMWR Morb Mortal Wkly Rep 73:149–151. doi:10.15585/mmwr.mm7307a3 38386615 PMC10899077

[18] Waites KB, Xiao L, Liu Y, Balish MF, Atkinson TP. 2017. Mycoplasma pneumoniae from the respiratory tract and beyond. Clin Microbiol Rev 30:747–809. doi:10.1128/CMR.00114-16 28539503 PMC5475226

[19] Kim K, Jung S, Kim M, Park S, Yang HJ, Lee E. 2022. Global trends in the proportion of macrolide-resistant Mycoplasma pneumoniae Infections: a systematic review and meta-analysis. JAMA Netw Open 5:e2220949. doi:10.1001/jamanetworkopen.2022.20949 35816304 PMC9274321

[20] Jiang Z, Li S, Zhu C, Zhou R, Leung PHM. 2021. Mycoplasma pneumoniae infections: pathogenesis and vaccine development. Pathogens 10:119. doi:10.3390/pathogens10020119 33503845 PMC7911756

[21] Zhang N, Zeng W, Du T, Wei H, Tian W, Meng Y, He G, Lei A, Zhu C. 2023. Lacticaseibacillus casei CNRZ1874 supplementation promotes M1 alveolar macrophage activation and attenuates Mycoplasma pneumoniae pneumonia. J Appl Microbiol 134:lxad022. doi:10.1093/jambio/lxad022 36731870

[22] Long H, He G, He J, Du TF, Feng P, Zhu C. 2024. The protective effect and immunomodulatory ability of orally administrated Lacticaseibacillus rhamnosus GG against Mycoplasma pneumoniae infection in BALB/c mice. PLoS One 19:e0312318. doi:10.1371/journal.pone.0312318 39453930 PMC11508164

[23] Wubbel L, Jafri HS, Olsen K, Shelton S, Barton Rogers B, Gambill G, Patel P, Keyser E, Cassell G, McCracken GH. 1998. Mycoplasma pneumoniae pneumonia in a mouse model. J Infect Dis 178:1526–1529. doi:10.1086/314439 9780280

[24] Chu HW, Breed R, Rino JG, Harbeck RJ, Sills MR, Martin RJ. 2006 a. Repeated respiratory Mycoplasma pneumoniae infections in mice: effect of host genetic background. Microbes Infect 8:1764–1772. doi:10.1016/j.micinf.2006.02.014B4 16713727

[25] Meyer Sauteur PM, de Groot RCA, Estevão SC, Hoogenboezem T, de Bruijn ACJM, Sluijter M, de Bruijn MJW, De Kleer IM, van Haperen R, van den Brand JMA, Bogaert D, Fraaij PLA, Vink C, Hendriks RW, Samsom JN, Unger WWJ, van Rossum AMC. 2018. The role of B cells in carriage and clearance of Mycoplasma pneumoniae from the respiratory tract of mice. J Infect Dis 217:298–309. doi:10.1093/infdis/jix559 29099932

[26] Chiba E, Tomosada Y, Vizoso-Pinto MG, Salva S, Takahashi T, Tsukida K, Kitazawa H, Alvarez S, Villena J. 2013. Immunobiotic Lactobacillus rhamnosus improves resistance of infant mice against respiratory syncytial virus infection. Int Immunopharmacol 17:373–382. doi:10.1016/j.intimp.2013.06.024 23838113

[27] Ma J, Rubin BK, Voynow JA. 2018. Mucins, mucus, and goblet cells. Chest 154:169–176. doi:10.1016/j.chest.2017.11.008 29170036

[28] Kim YC, Sohn KH, Kang HR. 2024. Gut microbiota dysbiosis and its impact on asthma and other lung diseases: potential therapeutic approaches. Korean J Intern Med 39:746–758. doi:10.3904/kjim.2023.451 39252487 PMC11384250

[29] Tamiya S, Yoshikawa E, Ogura M, Kuroda E, Suzuki K, Yoshioka Y. 2021. Neutrophil-mediated lung injury both via TLR2-dependent production of IL-1α and IL-12 p40, and TLR2-independent CARDS toxin after Mycoplasma pneumoniae infection in mice. Microbiol Spectr 9:e0158821. doi:10.1128/spectrum.01588-21 34937175 PMC8694186

[30] Erb P, Bredt W. 1979. Interaction of Mycoplasma pneumoniae with alveolar macrophages: viability of adherent and ingested mycoplasmas. Infect Immun 25:11–15. doi:10.1128/iai.25.1.11-15.1979 113340 PMC414413

[31] Hickman-Davis JM, Lindsey JR, Zhu S, Matalon S. 1998. Surfactant protein A mediates mycoplasmacidal activity of alveolar macrophages. Am J Physiol Lung Cell Mol Physiol 274:L270–L277. doi:10.1152/ajplung.1998.274.2.L270 9486213

[32] Guo L, Liu F, Lu MP, Zheng Q, Chen ZM. 2015. Increased T cell activation in BALF from children with Mycoplasma pneumoniae pneumonia. Pediatr Pulmonol 50:814–819. doi:10.1002/ppul.23095 25157471

[33] Chen Z, Shao X, Dou X, Zhang X, Wang Y, Zhu C, Hao C, Fan M, Ji W, Yan Y. 2016. Role of the Mycoplasma pneumoniae/interleukin-8/neutrophil axis in the pathogenesis of pneumonia. PLoS One 11:e0146377. doi:10.1371/journal.pone.0146377 26752656 PMC4708980

[34] Wang C, Wen J, Yan Z, Zhou Y, Gong Z, Luo Y, Li Z, Zheng K, Zhang H, Ding N, Wang C, Zhu C, Wu Y, Lei A. 2024. Suppressing neutrophil itaconate production attenuates Mycoplasma pneumoniae pneumonia. PLoS Pathog 20:e1012614. doi:10.1371/journal.ppat.1012614 39499730 PMC11567624

[35] Guo H, He Z, Li M, Wang T, Zhang L. 2017. Corrigendum to “Imbalance of peripheral blood Th17 and Treg responses in children with refractory Mycoplasma pneumoniae pneumonia” [J Infect Chemother 22 (2016) 162–166]. J Infect Chemother 23:193. doi:10.1016/j.jiac.2016.12.005 26806148

[36] Yang M, Meng F, Wang K, Gao M, Lu R, Li M, Zhao F, Huang L, Zhang Y, Cheng G, Wang X. 2017. Interleukin 17A as a good predictor of the severity of Mycoplasma pneumoniae pneumonia in children. Sci Rep 7. doi:10.1038/s41598-017-13292-5 PMC5636901 29021577

[37] Luo Y, Li C, Zhou Z, Gong Z, Zhu C, Lei A. 2021. Biological functions of IL-17-producing cells in mycoplasma respiratory infection. Immunology 164:223–230. doi:10.1111/imm.13346 33930194 PMC8442233

[38] Liu D, Tan Y, Bajinka O, Wang L, Tang Z. 2020. Th17/IL-17 axis regulated by airway microbes get involved in the development of asthma. Curr Allergy Asthma Rep 20:11. doi:10.1007/s11882-020-00903-x 32172346

[39] Wu Q, Martin RJ, Rino JG, Breed R, Torres RM, Chu HW. 2007. IL-23-dependent IL-17 production is essential in neutrophil recruitment and activity in mouse lung defense against respiratory Mycoplasma pneumoniae infection. Microbes Infect 9:78–86. doi:10.1016/j.micinf.2006.10.012 17198762 PMC1832075

[40] Kurata S, Osaki T, Yonezawa H, Arae K, Taguchi H, Kamiya S. 2014. Role of IL-17A and IL-10 in the antigen induced inflammation model by Mycoplasma pneumoniae. BMC Microbiol 14:156. doi:10.1186/1471-2180-14-156 24928272 PMC4074139

[41] Takahashi R, Shiohara T, Mizukawa Y. 2021. Monocyte-Independent and -dependent regulation of regulatory T-cell development in Mycoplasma infection. J Infect Dis 223:1733–1742. doi:10.1093/infdis/jiaa590 32946556

[42] Capurso L. 2019. Thirty years of Lactobacillus rhamnosus GG: a review. J Clin Gastroenterol 53 Suppl 1:S1–S41. doi:10.1097/MCG.0000000000001170 30741841

[43] Dumke R, Jacobs E. 2016. Antibody response to Mycoplasma pneumoniae: protection of host and influence on outbreaks? Front Microbiol 7:39. doi:10.3389/fmicb.2016.00039 26858711 PMC4726802

[44] Foy HM. 1993. Infections caused by Mycoplasma pneumoniae and possible carrier state in different populations of patients. Clin Infect Dis 17 Suppl 1:S37–46. doi:10.1093/clinids/17.supplement_1.s37 8399936

[45] Taylor G, Taylor-Robinson D, Fernald GW. 1974. Reduction in the severity of Mycoplasma pneumoniae-induced pneumonia in hamsters by immunosuppressive treatment with antithymocyte sera. J Med Microbiol 7:343–348. doi:10.1099/00222615-7-3-343 4547395

